# Novel Method to Load Multiple Genes onto a Mammalian Artificial Chromosome

**DOI:** 10.1371/journal.pone.0085565

**Published:** 2014-01-15

**Authors:** Anna Tóth, Katalin Fodor, Tünde Praznovszky, Vilmos Tubak, Andor Udvardy, Gyula Hadlaczky, Robert L. Katona

**Affiliations:** 1 Institute of Genetics, Biological Research Centre, Hungarian Academy of Sciences, Szeged, Hungary; 2 Institute of Biochemistry, Biological Research Centre, Hungarian Academy of Sciences, Szeged, Hungary; University of Szeged, Hungary

## Abstract

Mammalian artificial chromosomes are natural chromosome-based vectors that may carry a vast amount of genetic material in terms of both size and number. They are reasonably stable and segregate well in both mitosis and meiosis. A platform artificial chromosome expression system (ACEs) was earlier described with multiple loading sites for a modified lambda-integrase enzyme. It has been shown that this ACEs is suitable for high-level industrial protein production and the treatment of a mouse model for a devastating human disorder, Krabbe’s disease. ACEs-treated mutant mice carrying a therapeutic gene lived more than four times longer than untreated counterparts. This novel gene therapy method is called combined mammalian artificial chromosome-stem cell therapy. At present, this method suffers from the limitation that a new selection marker gene should be present for each therapeutic gene loaded onto the ACEs. Complex diseases require the cooperative action of several genes for treatment, but only a limited number of selection marker genes are available and there is also a risk of serious side-effects caused by the unwanted expression of these marker genes in mammalian cells, organs and organisms. We describe here a novel method to load multiple genes onto the ACEs by using only two selectable marker genes. These markers may be removed from the ACEs before therapeutic application. This novel technology could revolutionize gene therapeutic applications targeting the treatment of complex disorders and cancers. It could also speed up cell therapy by allowing researchers to engineer a chromosome with a predetermined set of genetic factors to differentiate adult stem cells, embryonic stem cells and induced pluripotent stem (iPS) cells into cell types of therapeutic value. It is also a suitable tool for the investigation of complex biochemical pathways in basic science by producing an ACEs with several genes from a signal transduction pathway of interest.

## Introduction

Gene therapy may be extremely promising for the treatment of various genetic and acquired disorders in the near future [1]. There is currently only one feature delaying the clinical success of this revolutionary technique: the lack of a safe, stable and reliable genetic vector. We believe that a carefully engineered mammalian artificial chromosome could fill this void as a genetic carrier [2]–[21].

Four different strategies have been developed for the construction of artificial chromosomes [22]: (i) in the synthetic approach the artificial chromosome is assembled from chromosomal components [23]–[33], (ii) the ‘top down’ method applies the *in vivo* telomere-associated fragmentation of existing chromosomes [34]–[45], (iii) naturally occurring minichromosomes may be engineered to construct an artificial chromosome [46], and (iv) de novo chromosome generation can be induced via targeted amplification of specific chromosomal segments [47]–[53]. To date, two technologies, the ‘top down’ and the induced de novo chromosome generation approaches have advanced to the point suitable for biotechnological applications. The artificial chromosome technology exploits the unique properties of artificial chromosomes as being engineered chromosomes with defined genetic contents, capable of functioning as non-integrating vectors with large carrying capacity and stability [13], [54].

The incorporation of different integrase recognition sequences and integrase enzyme genes into artificial chromosomes greatly improved the feasibility of integrating a transgene into the artificial chromosome [52], [55]–[57].

Recently an artificial chromosome expression system (ACEs) was constructed which carry multiple copies (>50) of recombination acceptor sites (attB) for multiple unidirectional loading of expression cassettes by a modified λ integrase [52]. Two different applications justify the power of this artificial chromosome expression system. Stable, high MAb expressing CHO cell lines were generated by the ACE technology under 6 months, which expressed 1 g/L of human monoclonal IgG1 antibody. The cell lines performed stable expression of the monoclonal antibody for up to 70 days during continuous culture [58], [59].

The recently developed novel method, in which the ACEs was combined with stem cell therapy for the treatment of a mouse model for a devastating human disorder, Krabbe’s disease [60], demonstrates the success of the mammalian artificial chromosome technology for gene therapy application. The limitation of this new technique is the requirement of repeated loading of target genes to the artificial chromosome for gene therapy of multi-gene genetic disorders. Although the multitudes of the recombination acceptor sites on the ACEs chromosome allow the repeated loading of different genes, selection of each newly loaded gene requires the presence of a different selection marker. We describe here a novel method through which to load multiple genes onto the ACEs by using only two selectable marker genes which may be removed from the ACEs before therapeutic application.

## Materials and Methods

### Plasmids

**Figure 1:**
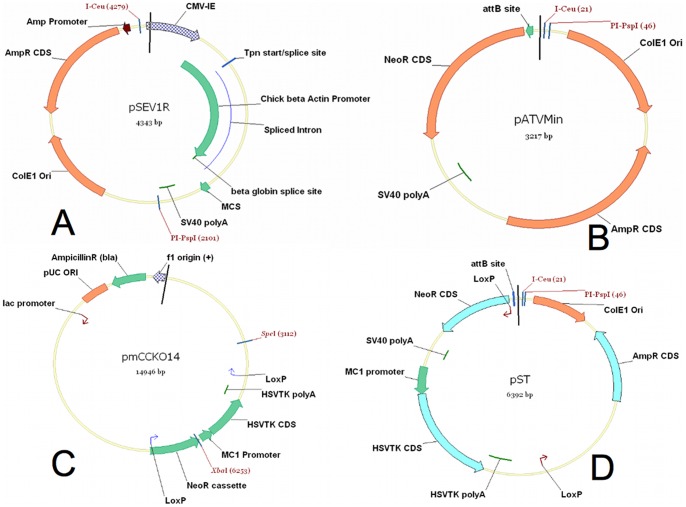
The pST plasmid vector was produced to achieve the superloading of Platform ACEs chromosomes. (A) The pSEV1R (shuttle expression vector-1R) entry vector map. The gene of interest is cloned into the multi-cloning site (MCS) of this vector. MCS is part of an expression cassette with a chicken beta-actin promoter and an SV40 polyA signal. The I-Ceu and PI-PspI restriction enzyme recognition sites flank the expression cassette. The whole expression cassette with the gene of interest can be moved from this plasmid into several different types of Platform ACEs targeting plasmids (ATVs) with the help of these enzymes. (B) A Platform ACEs-targeting plasmid, pATVMin. The I-Ceu and PI-PspI enzyme sites are used to transfer the expression cassette with the gene of interest into pATVMin. The attB site is the recognition site of ACE integrase. This enzyme performs site-specific recombination between the attB site and the attP site found on Platform ACEs. This process integrates the pATVMin plasmid together with the gene of interest in the expression cassette into the artificial chromosome. After integration, the promoterless neomycin resistance gene acquires the promoter of puromycin gene on the Platform ACEs and G418-resistant cell lines can be isolated with ACE chromosomes carrying and expressing the gene of interest. (C) The pmccKO14 plasmid map. The HSV-TK expression cassette with the LoxP site was removed from this plasmid by XbaI-SpeI restriction enzyme digestion and transferred into the pATVMin plasmid. (D) The map of the pST plasmid. This plasmid contains a promoterless neomycin gene and a HSV-TK expression cassette flanked by direct repeats of two LoxP sites. The HSVT-TK expression cassette came into this plasmid from the pmccKO14 vector by ligation into the unique XbaI site in the pATVMin plasmid. The LoxP site upstream from NeoR CDS was inserted by a PCR-based method. The gene of interest is transferred into the pST vector by the method described in (B). This transgene-carrying vector is then loaded onto the Platform ACEs chromosome as described in (B) and previously published^7^. The LoxP-flanked selectable marker gene cassette can be removed by the transient action of Cre recombinase. Through the use of ganciclovir selection, cell lines lacking the neomycin-thymidine kinase selectable marker gene cassette can be isolated.

**Figure 2:**
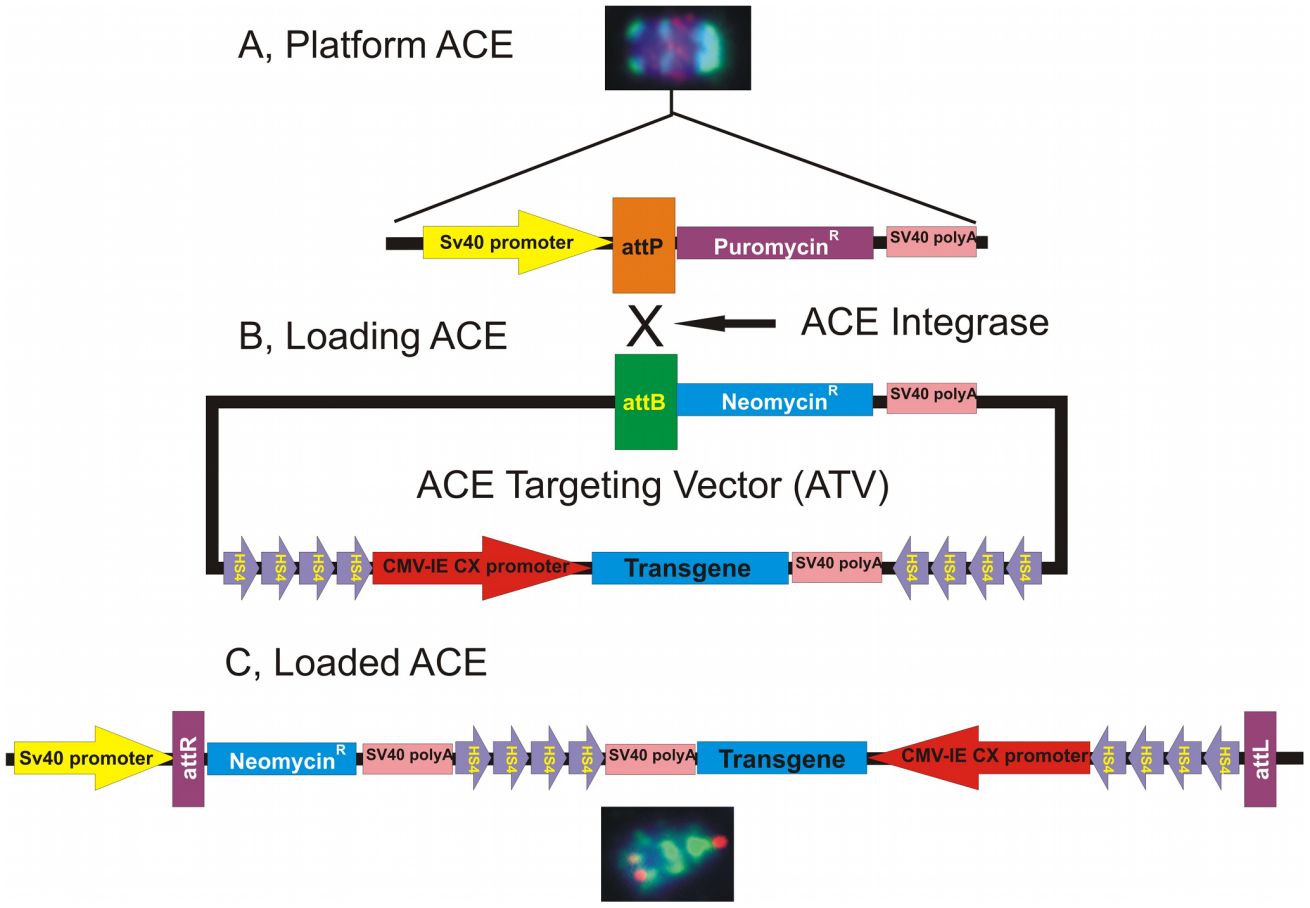
The schematics of the site-specific integration process by which the ACE chromosome was targeted with therapeutic transgenes. (A) The Platform ACEs contains multiple attP recombination acceptor sites for the ACE integrase. The attP site is situated between an SV40 promoter and the puromycin resistance gene, which is driven by this promoter. A Platform ACEs chromosome is also shown. The chromosome is counterstained by DAPI (blue). The green fluorescent staining demonstrates the presence of mouse major satellite sequences, major components of the ACE chromosome. Red fluorescent staining designates the attP sequence carrying sites suitable for the site-specific integration of transgenes. (B) The ATV vectors carry the attB recombination site for the ACE integrase. There is a promoterless neomycin resistance gene immediately after the attB site. The ACE integrase catalyzes the site-specific recombination between the attB and attP sites and integrates the ATV vector into the ACE chromosome. (C) The integration event disconnects the puromycin resistance gene from its promoter and replaces it with the promoterless neomycin resistance gene, which acquires the SV40 promoter. Targeted, transgene-carrying cell lines can be selected for G418 resistance. A transgene-loaded ACE chromosome is also present at the bottom of this figure. The green fluorescent staining demonstrates the presence of mouse major satellite sequences, major components of the ACE chromosome. Red fluorescent staining demonstrates the presence of the “loaded” transgene on the ACE chromosome. The chromosome is counterstained by DAPI (blue).

The pSEV1R (shuttle expression vector-1R) entry vector (Figure 1A) and the pATVMin ATV vector (Figure 1B) were constructed and kindly donated by Chromos Molecular Systems Inc. (now Calyx Bio-Ventures Inc., www.calyxbio.com). The pATVMin vector was previously used to load transgenes onto Platform ACEs as shown on Figure 2. The pST plasmid was constructed from the pATVMin plasmid as follows: the pATVMin plasmid was digested with BamHI restriction endonuclease. The LoxP sequence-containing oligos were synthetized with overhanging BglII restriction sites (LoxpBGF: 5′- GATCTataacttcgtataatgtatgctatacgaagttatA, LoxPBGR: 5′- GATCTataacttcgtatagcatacattatacgaagttatA). Double-stranded oligos were produced from these single-stranded oligos by standard methods. The double-stranded LoxP oligo was ligated into the BamHI site of the pATVMin plasmid. Correct orientation of the LoxP site was checked by DNA sequencing. We earlier constructed a plasmid vector, pmCCKO14 (unpublished result; Figure 1C). This vector carried a *Herpes simplex* virus thymidine kinase (HSVTK) expression cassette, flanked by a LoxP site downstream from the polyA signal. The LoxP site carrying pATVMin plasmid was digested with XbaI. The thymidine kinase expression cassette with the LoxP site was removed from the pmCCKO14 plasmid as an XbaI-SpeI fragment. This fragment was cloned into the LoxP site-containing, XbaI enzyme-digested pATVMin vector. The resulting plasmid was checked by DNA sequencing. The plasmid with the correct sequence was named pST (Figure 1D).

**Figure 3:**
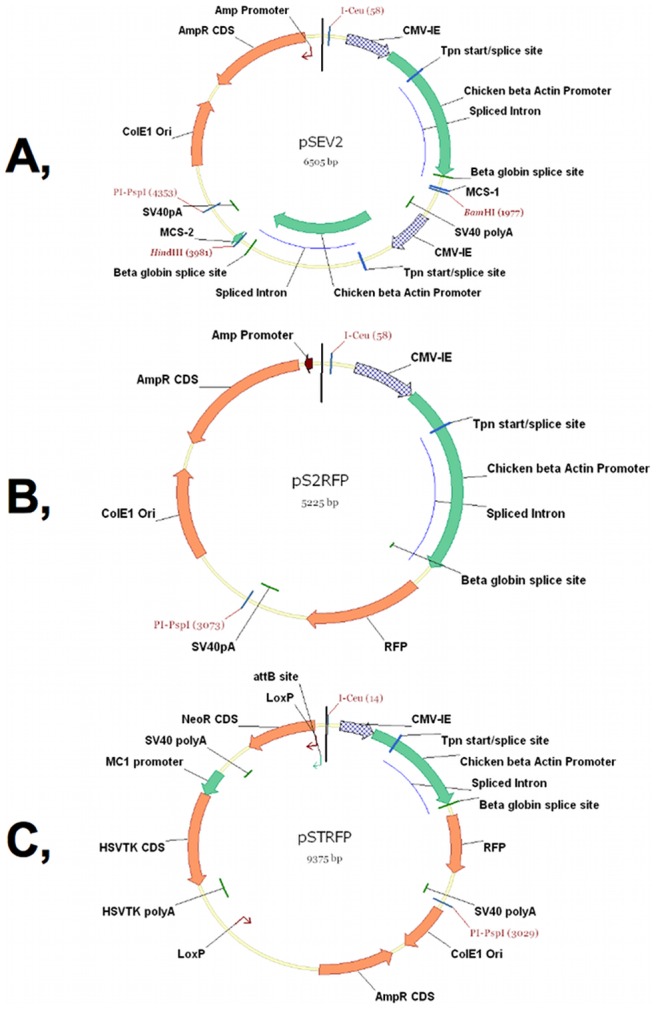
The assembly of the pSTRFP plasmid. (A) The pSEV2 entry vector was digested with the BamHI and HindIII restriction enzymes. (B) The pmR-mCherry plasmid was digested with the BamHI and HindIII restriction endonucleases, the RFP CDS-containing DNA fragment was isolated and inserted into the pSEV2 plasmid and the pS2RFP vector was assembled. (C) The I-Ceu and PI-PspI enzymes were used to transfer the mCherry expression cassette from pS2RFP into the pST plasmid and the pSTRFP ACE targeting vector was produced.

**Figure 4:**
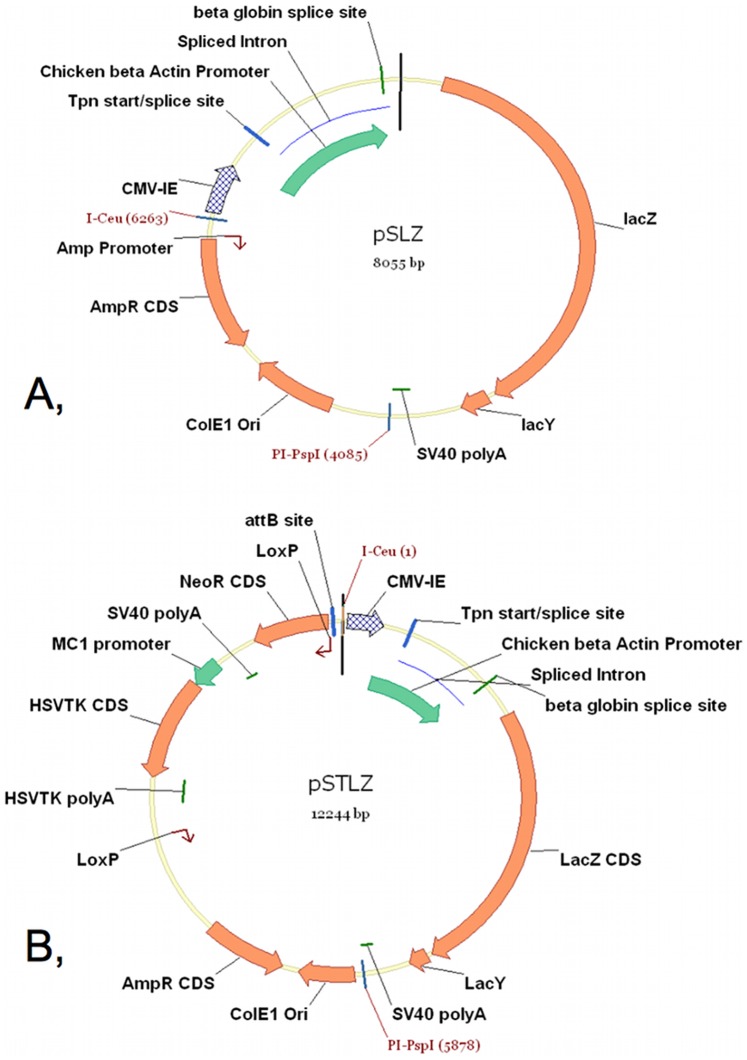
The production of the pSTLZ plasmid. (A) The pCH110 (Amersham) plasmid was digested with the BamHI and HindIII restriction endonucleases and the LacZ-LacY-containing DNA fragment was isolated. The pSEV1R plasmid (Figure 1A) was digested with the BglII and HindIII restriction enzymes. The appropriate DNA fragment was isolated and the LacZ-LacY was ligated together with this fragment to yield the pSLZ plasmid. (B) The LacZ-LacY expression cassette was removed from pSLZ plasmid with the I-Ceu and PI-PspI homing endonucleases. Subsequently, this cassette was ligated into the pST vector, previously digested with the same endonucleases, and the pSTLZ plasmid was constructed.

The pSTRFP plasmid was constructed by cloning the coding sequence of the mCherry gene into the pST plasmid vector (from pmR-mCherry, Clontech, 632542) as follows: the pmR-mCherry plasmid was digested with the BamHI and HindIII. The mCherry CDS (in the plasmid construct we call this briefly RFP) containing DNA fragment was isolated. The pSEV2 (shuttle expression vector-2) entry vector was constructed and kindly donated by Chromos Molecular Systems Inc. (now Calyx Bio-Ventures Inc., www.calyxbio.com) (Figure 3A). This vector was digested with BamHI and HindIII. The appropriate DNA fragment was isolated and the RFP CDS was ligated together with this fragment. As a result, the pS2RFP plasmid was obtained (Figure 3B). The RFP expression cassette was removed from this plasmid with the I-Ceu and PI-PspI homing endonucleases. Subsequently, this cassette was ligated into the pST vector, previously digested with the same endonucleases, and the pSTRFP plasmid was constructed (Figure 3C). The pSTLZ plasmid was produced by cloning the coding sequence of the beta-galactosidase gene into the pST plasmid vector (from pCH110, Amersham) as follows: the pCH110 plasmid was digested with BamHI and HindIII and the LacZ-LacY-containing DNA fragment was isolated. The pSEV1R vector was digested with BglII and HindIII. The appropriate DNA fragment was isolated and the LacZ-LacY was ligated together with this fragment. As a result, the pSLZ plasmid was obtained (Figure 4A). The LacZ-LacY expression cassette was removed from this plasmid with the I-Ceu and PI-PspI homing endonucleases. Subsequently, this cassette was ligated into the pST vector, which was previously digested with the same endonucleases, and the pSTLZ plasmid was constructed (Figure 4B). The pCre-GFP plasmid (a gift from Jim Downing’s laboratory, St. Jude Children’s Research Hospital, Memphis, TN, USA) was used for the transient expression of Cre recombinase to remove the neomycin-thymidine kinase selectable marker gene cassette flanked by direct LoxP sites from the ACEs chromosomes. The pCXLamIntROK plasmid vector was used to transiently express ACE integrase for site-specific loading of transgenes onto the ACE chromosomes. This plasmid was kindly donated by Chromos Molecular Systems Inc. (map is not shown, now Calyx Bio-Ventures Inc., www.calyxbio.com).

### Cell Cultures

The Platform ACEs-carrying Y2913D-SFS Chinese hamster ovary (CHO DG44) cell line from Chromos Molecular Systems Inc. was cultured in MEM alpha (Gibco, 22571), 5% FCS, streptomycin-penicillin (Gibco, 15070–063) and 10 mg/ml puromycin (Sigma, P-7255). The 1D9-16, RFPG18 and RFPG18-12 cell lines were generated in our laboratory and their properties are described in detail in the Results and Discussion section. These cell lines were cultured in the same medium as described above except that the puromycin was omitted. The 1D9-16 and RFPG18-12 cell lines were cultured in the presence of 400 µg/ml G418 (Sigma, G-5013). The RFPG18 cell line was cultured in the presence of 10 µM ganciclovir (Sigma, G-2536).

### Cell Transfection

Plasmid transfection experiments were performed with the Superfect reagent (Qiagen, 301305) as described by the manufacturer.

### FISH

Fluorescence *in situ* hybridization (FISH) experiments were performed with a standard protocol. The pPur (Clontech, 631601) plasmid was labeled with FITC fluorescent dye and used as a probe for ACE detection. We used the Roche DIG Nick Translation Mix (1745816) or the Roche Biotin Nick Translation Mix (1745824) for labeling DNA probes.

### PCR

Genomic DNA samples were isolated with the Zymo Research Quick-gDNA MiniPrep (Cat. No.: D3025). Site-specific integration of transgenes into the ACE chromosome was detected by PCR experiments with the following primers: 193AF: 5′-ACCCCCTTGCGCTAATGCTCTGTTA and NeoR1 5′-TCGATGAATCCAGAAAAGCGGCCA. The expected product size is 784 bp.

### Microscopy

All pictures were photographed under a Zeiss Axiovision Z1 fluorescent microscope by using the Axiovision software purchased with the microscope.

## Results and Discussion

A Platform ACEs chromosome with multiple recombination recognition sites for a special lambda-integrase (ACE integrase) was earlier constructed and a vector system was additionally established to deliver “useful genes” onto the Platform ACEs [52]. This system begins with an entry vector (Figure 1A; pSEV1R), which contains an expression cassette including a multiple cloning site for the insertion of a gene of interest, or briefly transgene [52]. Subsequently, this expression cassette is introduced into an ACE-targeting vector (ATV) by the action of the yeast homing endonucleases I-Ceu and PI-PspI (Figure 1B; pATVMin). The recognition sites of these enzymes flank the entire transgene expression cassette in the entry vector. These sites are also found in the ATV. This system ensures that a transgene expression cassette can be delivered from one entry vector into several different types of ATV plasmids. These ATV plasmids carry a mammalian selectable marker gene (neomycin) without a promoter (examples are shown in Figure 1B, D). These vectors harbor the recombination recognition site for the ACE integrase just upstream from the translation start of the mammalian selectable marker gene (Figure 1B, D). The ATV harboring the gene of interest is then co-transfected into a Platform ACEs carrying cell line with a plasmid that transiently expresses the ACE integrase (Figure 2A, B). The ACE integrase catalyzes recombination between its recognition site on the Platform ACEs (attP) and on the ATV (attB, Figure 2B). This recombination is unidirectional: the ATV integrates into the ACE chromosome by a site-specific recombination event. This event results in the “loaded” ACE chromosome, where the gene of interest is built into the Platform ACEs (Figure 2C). We are able to select for this event, as the promoterless neomycin marker gene acquires a promoter on the artificial chromosome (the SV40 promoter of the puromycin gene, Figure 2A) and the “loaded” Platform ACEs chromosome-carrying cell lines become G418-resistant. In general, we refer to these “loaded” Platform ACEs chromosomes as therapeutic Artificial Chromosomes (tACs). Although it harbors about 50 to 200 loading sites for gene delivery, the number of useful genes that can be loaded onto the presently available Platform ACEs is limited to the selectable marker genes that are available and that number is very small. In many cases, this can be a problem, as the loading of multiple genes onto the ACE chromosome might be important (e.g.: in transgenesis experiments with multiple genes, for the examination and understanding of biochemical pathways, for the development of therapeutic applications for cancer and complex diseases, etc.). Moreover, some of the selectable marker genes may have unwanted side-effects when they are expressed in elevated levels in certain tissues and organs in the body. This restricts the therapeutic and scientific applications of the Platform ACEs.

**Figure 5:**
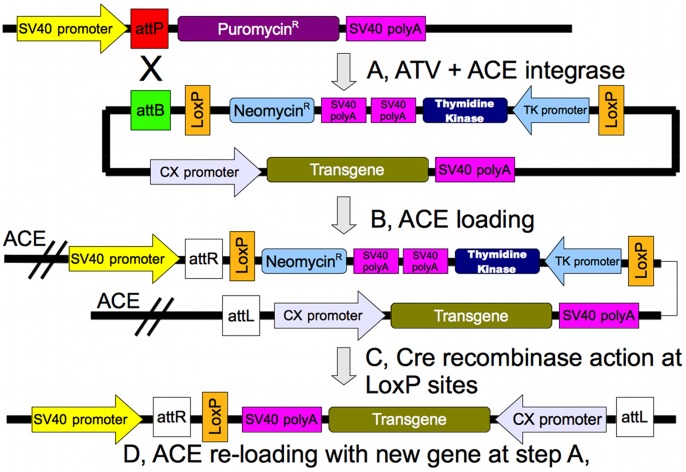
The “superloading” cycle. The transgene is cloned into the pST plasmid and loaded onto the ACE chromosome as shown in Figure 1. In the third step the Cre recombinase is transiently expressed in the cell line carrying the transgene-loaded ACE. The Cre recombinase removes the neomycin-thymidine kinase selectable marker gene cassette found between the direct repeats of LoxP sites. Transgene-loaded ACE chromosome-carrying cell lines that lack the neomycin-thymidine kinase selectable marker gene cassette can be obtained by ganciclovir selection. The ACE chromosome is now suitable for the loading of a new transgene cloned into the pST plasmid vector.

We report here the development of a new vector system, which makes use of only one selectable marker gene cassette for the delivery of a transgene (Figure 1D). Subsequently, this vector may be recycled and used for the loading of new genes. The novel selectable marker gene cassette consists of a promoterless neomycin gene (NeoR) just downstream from the ACE integrase recognition site and a thymidine kinase (HSVTK) expression cassette, which is equipped with an MC1 promoter and an HSVTK polyadenylation signal (Figure 1D). LoxP sites in direct orientation flank the NeoR and HSVTK selectable marker gene cassette (Figure 1D). LoxP is the recognition site of the site-specific recombination enzyme, Cre. Cre is able to catalyze recombination between these two LoxP sites, and as a result the whole selectable marker gene cassette is removed. This phenomenon is exploited in our gene loading system. This new type of ATV plasmid is referred to as a superloading vector (pST, Figure 1D) and an overview of the superloading cycle is presented in Figure 5.

**Figure 6:**
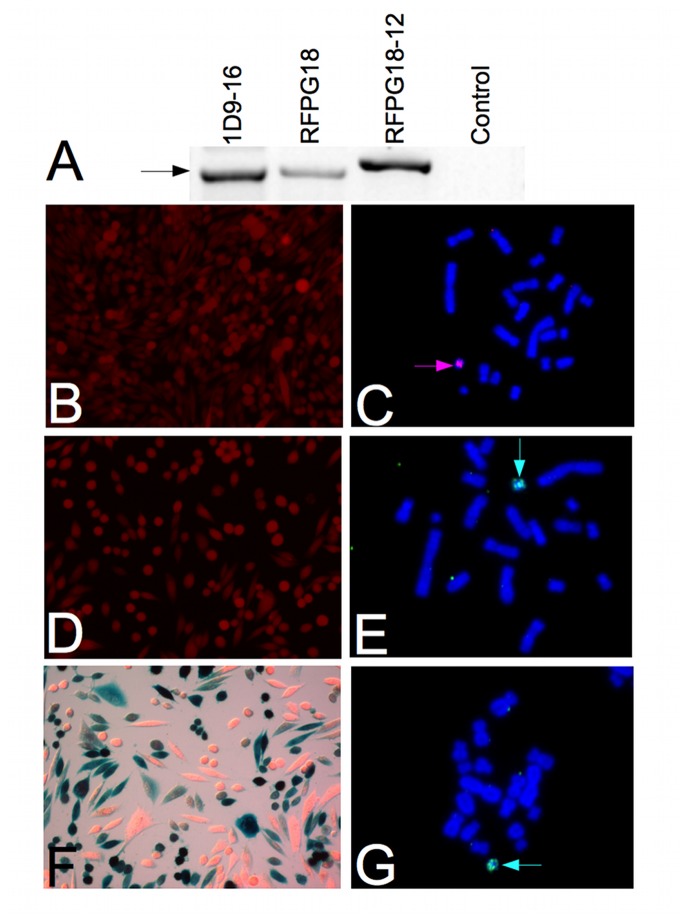
Experimental demonstration of the superloading process. (A) Site-specific integration of the ATV plasmid carrying the various transgenes is demonstrated by means of PCR experiments on purified genomic DNA samples. All the relevant transgene targeted cell lines are presented (1D-9-16, RFPG18 and RFPG18-12), each of which was positive for site-specific integration of the transgene, as demonstrated by the presence of the correct-sized PCR fragment (784 bp). (B) 1D9-16 cells emit the red fluorescence of the mCherry protein, expressed from the loaded ACE chromosome. (C) 1D9-16 cells carry the mCherry-loaded ACE chromosome, demonstrated by means of FISH experiments with a TRITC-labeled plasmid probe (magenta signal, magenta arrow). All chromosomes are counterstained with DAPI (blue signal) (M: 630x). (D) RFPG18 cells emit the red fluorescence of the mCherry protein, expressed from the loaded ACE chromosome (M: 200x). This cell line was treated with the transiently expressed Cre recombinase. The Cre protein removed the selection marker gene cassette (NeoTK) and left the mCherry expression cassette intact. The ACE is now suitable for re-loading with a new transgene. (E) The RFPG18 cells carry the mCherry-loaded ACE chromosome, demonstrated by means of FISH experiments with an FITC-labeled plasmid probe (green signal, green arrow). All chromosomes are counterstained with DAPI (blue signal) (M: 630x). (F) The RFPG18-12 cells emit the red fluorescence of the mCherry protein, expressed from the loaded ACE chromosome (M: 200x). The LacZ staining procedure also stains the RFPG18-12 cells blue. These results demonstrate that the second loading with beta-galactosidase was successful and beta-galactosidase is expressed from the superloaded ACE chromosome and functional (M: 200x). (G) The RFPG18-12 cells carry the mCherry and beta-galactosidase-loaded ACE chromosome, as demonstrated by means of FISH experiments with an FITC-labeled plasmid probe (green signal, green arrow). All chromosomes are counterstained with DAPI (blue signal) (M: 630x).

The first gene of interest is cloned into the superloading vector through the steps described in the Materials and Methods. For the proof of concept experiment reported, we chose the coding sequence of mCherry red fluorescent protein (*Discosoma sp.*) as the first gene to load onto the Platform ACEs and constructed the pSTRFP superloading vector (Figure 3C). This vector was then transfected into the Platform ACEs-carrying cell line along with the ACE integrase-expressing vector (pCXLamIntROK). Twenty-four hours later, antibiotic selection was started by G418 to acquire cell colonies carrying the superloading vector correctly targeted onto the Platform ACEs. A PCR experiment was used to verify site-specific integration (1D9-16 cell line; Figure 6A). PCR reactions were performed on purified genomic DNA samples originating from G418-resistant cell lines. Cell lines were additionally examined by fluorescent microscopy for the presence of the red fluorescent signal emitted by the mCherry protein (Figure 6B). We also investigated the new ACE chromosomes for the presence of the transgene on the intact ACE by FISH analysis (Figure 6C; magenta signal). Based on correct ACE targeting, a good fluorescent signal and a good-sized, intact ACE chromosome, we chose a single cell line (1D9-16) for further experiments (Figure 6A–C). This first step of the superloading cycle is presented graphically in Figure 5A, B. Ganciclovir, a synthetic analog of 2′-deoxy-guanosine, is first phosphorylated to a deoxyguanosine triphosphate (dGTP) analog by the viral thymidine kinase delivered onto the ACE by the pSTRFP vector. This competitively inhibits the incorporation of dGTP by DNA polymerase, resulting in the termination of elongation of DNA replication, which eventually results in cell death. The minimum effective concentration of ganciclovir that was sufficient to kill 1D9-16 cells was determined to be 10 µM. A Cre recombinase-expressing plasmid (pCre-GFP) was transfected into the 1D9-16 cells and Cre recombinase was transiently expressed (Figure 5C). Since the neomycin and thymidine kinase expression cassette is flanked by LoxP sites in direct orientation, we expected that Cre recombinase would effectively remove this marker gene construct from the ACE chromosome leaving behind only the gene of interest, in this case mCherry. Twenty-four hours after transfection, ganciclovir selection was started at 10 µM and resistant cell lines were isolated. Correctly targeted (Figure 6A; RFPG8-12), red fluorescence-emitting cell lines (Figure 6D) were further examined by FISH experiments (Figure 6E; green signal) for the presence of an intact ACE chromosome, as previously described. The RFPG18 cell line was chosen for further experiments. This point corresponded to the end of the first cycle of superloading. The ACE chromosome was now available for the loading of a new transgene (Figure 5D) and we therefore constructed a new superloading vector containing the coding sequence for the bacterial beta-galactosidase gene (LacZ) (see in Materials and Methods and Figure 4A, B). This new DNA construct, called pSTLZ (Figure 4B), carries the same selectable marker gene cassette flanked by direct repeats of LoxP sites as the pSTRFP vector. The pSTLZ plasmid was co-transfected with the ACE integrase-expressing plasmid into the RFPG18 cell line. This point marks the first step of the second loading cycle (Figure 5A, B). G418-resistant cell lines were isolated. Correct ACE targeting was determined by means of site-specific PCR experiments (Figure 6A; RFPG18-12). Cell lines were also tested for the positivity of LacZ staining and red fluorescence (Figure 6F), and were further analyzed for intact ACE chromosomes by FISH analysis (Figure 6G; green signal).

To analyze the efficiency of the repeated gene targeting procedure 68 stably transfected cell lines were generated after loading the ACEs with pSTRFP. 38 of these cell lines were positive for RFP signal (55%). Twenty five cell lines had proper targeting into the ACEs without random integration (36%) and all of these cell lines expressed the RFP protein. Based on strong RFP signal and intact ACEs, we have chosen the 1D9-16 cell line for further experiments.

Cre recombinase was transiently expressed in the 1D9-16 cell line to remove the neomycin-thymidine kinase selectable marker gene cassette flanked by direct LoxP sites from the ACEs chromosomes. Forty eight subclones were obtained with 10 µM ganciclovir selection. Twenty two cell lines (45%) which remained G418 resistant were discarded. The cell lines from which the neomycin-thymidine kinase selectable marker gene cassette was successfully removed were G418 sensitive and ganciclovir resistant. Twenty two cell lines (45%) carried intact ACEs and expressed the RFP protein. Based on strong RFP signal and intact ACEs, we have chosen the RFPG18 cell line for further experiments. The ACEs in the RFPG18 cell line was targeted with the pSTLZ vector (second loading of the ACEs). The LacZ gene was loaded onto the ACEs that already carried the RFP gene from the previous loading experiment. Seventy four G418 resistant cell lines were obtained. Thirty seven cell lines were positive for both RFP signal and LacZ staining (50%). Twenty one cell lines carried the LacZ and RFP genes exclusively on the ACEs (56.7%).

We identified cell lines with intact ACE chromosomes carrying and functionally expressing both mCherry and LacZ genes correctly targeted onto the artificial chromosome (Figure 6F, G). In these experiments, only one selectable marker gene cassette was used to deliver two different useful genes onto the ACE chromosome. The procedure presented here is easily fulfilled, highly efficient, requires minimal labor and presents no serious toxicity to the cells. Further, there is a possibility to remove all the selectable marker gene copies used to deliver useful genes onto the ACE in the last step before therapeutic application. This reduces the risk of side-effects due to the expression of genes coding for antibiotic resistance in the organism of a possible future patient.
